# Notch and Wnt Signaling Mediated Rod Photoreceptor Regeneration by Müller Cells in Adult Mammalian Retina

**DOI:** 10.1371/journal.pone.0012425

**Published:** 2010-08-26

**Authors:** Carolina Beltrame Del Debbio, Sudha Balasubramanian, Sowmya Parameswaran, Anathbandhu Chaudhuri, Fang Qiu, Iqbal Ahmad

**Affiliations:** 1 Department of Ophthalmology and Visual Sciences, College of Public Health, University of Nebraska Medical Center, Omaha, Nebraska, United States of America; 2 Department of Biostatistics, College of Public Health, University of Nebraska Medical Center, Omaha, Nebraska, United States of America; Boston University School of Medicine, United States of America

## Abstract

**Background:**

Evidence emerging from a variety of approaches used in different species suggests that Müller cell function may extend beyond its role of maintaining retinal homeostasis to that of progenitors in the adult retina. Enriched Müller cells *in vitro* or those that re-enter cell cycle in response to neurotoxin-damage to retina *in vivo* display multipotential and self-renewing capacities, the cardinal features of stem cells.

**Methodology/Principal Findings:**

We demonstrate that Notch and Wnt signaling activate Müller cells through their canonical pathways and that a rare subset of activated Müller cells differentiates along rod photoreceptor lineage in the outer nuclear layer. The differentiation of activated Müller cells along photoreceptor lineage is confirmed by multiple approaches that included Hoechst dye efflux analysis, genetic analysis using retina from Nrl-GFP mice, and lineage tracing using GS-GFP lentivirus in wild type and *rd* mice *in vitro* and S334ter rats *in vivo*. Examination of S334ter rats for head-neck tracking of visual stimuli, a behavioral measure of light perception, demonstrates a significant improvement in light perception in animals treated to activate Müller cells. The number of activated Müller cells with rod photoreceptor phenotype in treated animals correlates with the improvement in their light perception.

**Conclusion/Significance:**

In summary, our results provide a proof of principle for non-neurotoxin-mediated activation of Müller cells through Notch and Wnt signaling toward the regeneration of rod photoreceptors.

## Introduction

Müller cells are the sole glia generated by retinal stem cells/progenitors. Similar to the temporal pattern of generation of glia elsewhere in the central nervous system (CNS), Müller cells are born during the late stages of retinal histogenesis when the majority of neuronal cell types are already in the process of generation [1]. Therefore, progenitors during late histogenesis face the decision either to differentiate into neurons or glia. The mechanism through which such a decision is made is not clear, but the emerging evidence points towards Notch signaling. For example, if *Hes1* or *Hes5*, mediators of the canonical Notch pathway, is knocked out, Müller glia are either absent or present in a remarkably reduced number [2], [3] and if Notch signaling is decreased, Müller cell differentiation is adversely affected [4], [5], [6]. Interestingly, Notch signaling plays an important role in the maintenance of stem cells/progenitors throughout retinal histogenesis [6], [7], suggesting that it acquires a gliogenic role during Müller cell differentiation, presumably through the interactions with the JAK-STAT pathway [8], [9], [10].

Evidence emerging from a variety of approaches in different species suggests that Müller cell function may extend beyond its role of maintaining retinal homeostasis to that of a progenitor in the adult retina. In such a role, these cells are similar to their radially oriented cousin in the developing CNS, the radial glia, which subserve the function of stem cells [11], [12], [13], [14]. For example, in the mammalian system, enriched Müller cells *in vitro* and those that re-enter cell cycle in response to neurotoxin-damage *in vivo* display multipotential and self-renewing capacities, the cardinal features of stem cells [13]. In teleosts, where the retina regenerates throughout life and where the neurogenic property of Müller cells was initially recognized, it has been convincingly demonstrated that genetically tagged Müller cells re-enter the cell cycle and differentiate into retinal neurons at the site of the injury [14], [15], [16]. Examination of neurotoxin-damaged chick or mammalian retina revealed a rare subset of activated Müller cells tagged with BrdU or a genetic marker, expressing markers corresponding to retinal neurons [17], [18], [19], [20], [21]. In another approach, prospectively enriched Müller stem cells upon transplantation incorporated into host retina and differentiated along photoreceptor lineage, providing direct proof of the neurogenic property Müller cells in mammals [13].

These studies demonstrated that the neurogenic property of Müller cells is evolutionarily conserved and may be activated for the regeneration of the mammalian retina. Here, we provide a proof of principle for non-neurotoxin-mediated activation of Müller cells through Notch and Wnt signaling toward the regeneration of photoreceptors. We demonstrate, first in the explants of wild type mouse retina, that Notch and Wnt signaling activate Müller cells through their canonical pathways and that a rare subset of the activated Müller cells, identified by incorporated BrdU and expression of Müller cell-specific markers, differentiate along rod photoreceptor lineage in the outer nuclear layer. The differentiation of activated Müller cells along the photoreceptor lineage was confirmed by multiple approaches that included Hoechst dye efflux analysis [13], genetic analysis using the retina from Nrl-GFP mice [22], and lineage tracing using GS-GFP lentivirus [23]. Activation of Notch and Wnt signaling in explants of *rd* mice retina *in vitro* and S334ter retina *in vivo* before the onset of degeneration similarly led to activation of Müller cells and a rare subset of activated Müller cells were observed expressing rod photoreceptor-specific markers in the degenerated outer nuclear layers. Examination of S334ter rats for head-neck tracking of visual stimuli, a behavioral measure of light perception, demonstrated a significant improvement in light perception in animals treated to activate Müller cells and the number of activated Müller cells correlated with the improvement in the light perception. In summary, our results suggest that neurotoxin-free activation of Müller cells via Notch and Wnt signaling toward functional regeneration of rod photoreceptors is a possibility and its practical application will directly depend upon increasing the efficiency of converting Müller cells into rod photoreceptors.

## Materials and Methods

This study was approved by the Institutional Animal Care and Use Committee (IACUC), at University of Nebraska Medical Center (protocols #97-100-08FC and #95-005-09FC), and animals were housed and bred in the Department of Comparative Medicine at University of Nebraska Medical Center.

### Explant and Neurosphere Culture

Eyes from C57BL/6 (Jackson Laboratory, Maine, USA), and Nrl-GFP [22] mice at postnatal day 21 (PN21), and PN11 C3H/HeJ (rd) mice (Jackson Laboratory Maine, USA) were enucleated. Retina from each eye was removed, placed with retinal ganglion cell layer (GCL) side up on a semi-permeable membrane (0.4 µm pore size; Millipore, Temecula, California, USA), and cultured in RCM (DMEM-F12, N2 supplement, 2 mM L-glutamine, 100 U/ml penicillin, 100 g/ml streptomycin), 5% fetal bovine serum (FBS, Hyclone), and BrdU (50 µM, Sigma), for four days. Culture medium was supplemented with Jagged1 peptide (Jag1) or with scrambled sequence (Jag1^scrambled^, New England Peptide, Gardner, MA) and DAPT (Sigma, St. Lois, MO) to activate or inhibit Notch signaling, respectively. Wnt3a (R&D Systems, Minneapolis, MN) was added to activate Wnt signaling, and was neutralized by incubation with FzdCRD conditioned medium (CM) [13], [23]. Concentrations of reagents for each experiment are given in figures or figures legend. Explants were washed to remove traces of BrdU, and cultured in the presence of RCM +5% FBS +50% PN1 retinal conditioned medium (PN1 CM) to promote rod photoreceptor differentiation [13]. TUNEL assay (Promega, Madison, WI) and/or cleaved Caspase3 immunostaining were carried out on explant sections to determine cell viability (**Fig. S1**). Neurosphere assay was carried out as previously described [13]. Briefly, enriched Müller cells were cultured in 96 wells plate (0.32 cm^2^ area) in the presence of RCM + EGF (20 ng/ml, R&D Systems, Minneapolis, MN), for 4-5 days. Treatment to activate Notch and Wnt signaling and controls are the same as described above.

### Intravitreal injections

PN10 S334ter rats were anesthetized (Ketamine 12 mg/100 g of body weight, and Xylazine 0.8 mg/100 g of body weight) and a cocktail of Wnt2b CM [13], [24], [25], Jag1 (20 µM) and BrdU (1 µg/eye) was injected intravitreally to activate Müller cells. To promote differentiation along the rod photoreceptor lineage, a cocktail of PN1 CM, Shh (150 µg/µl, R&D Systems, Minneapolis, MN), and DAPT (30 µM) was injected intravitreally. The scheme of injections during the activation and differentiation phases is given in Figure 12 (A).

### Hoechst dye efflux assay

Retinal explants at the end of activation (4^th^ day), and differentiation (8^th^ day) phases were subjected to the Hoechst dye efflux assay, as previously described [13]. Briefly, cell dissociates incubated over night in Iscove's modified Dulbecco's medium (IMDM) and 2% FBS (10^6^ cells/ml), were stained with Hoechst 33342 (5 µg/ml) at 37°C for 30 minutes. Cells were sorted on a FACStar Plus (BD Biosciences, San Diego) cell sorter. The side population (SP) and non-side population (NSP) regions were defined on the basis of its fluorescence emission in both blue and red wavelengths. Dead cells and debris were excluded by establishing a live gate on the flow cytometer using forward versus side scatter. SP and NSP sorting gates were established after collecting 5×10^5^ events within this live gate.

### Polymerase Chain Reaction

Total RNA was isolated from the cells or explants using the MiniRNeasy Kit (Qiagen, Hilden, Germany) and cDNA was synthesized as previously described [26]. Specific transcripts were amplified with gene-specific forward and reverse primers using a step cycle program on a RoboCycler (Stratagene, La Jolla, CA) or Quantifast SYBR Green PCR kit (Qiagen), on a RotorGene 6,000 (Corbett Robotics, San Francisco, CA). The gene-specific primers used for RT-PCR and Q-PCR are described in Table 1. Semi-quantitative PCR products were resolved on 2% agarose gels. Quantitative PCR measurements were performed in triplicate; a reverse-transcription-negative blank of each sample and a no-template blank served as negative controls. Amplification curves and gene expression were normalized to the housekeeping gene GAPDH, used as an internal standard.

**Table 1:** List of primers.

Gene	Sequence	Size(bp)	T°	Accession No.
Ki67	F: 5′-GAGCAGTTACAGGGAACCGAAG -3′ R: 5′-CCTACTTTGGGTGAAGAGGCTG-3′	261	58	XM_001479849
Ki67	F: 5′-CAGCAGAAGAATCGTGGGAGAC -3′ R: 5′-CCTACTTTGGGTGAAGAGGTTGC-3′	103	54	XM_225460.4
CyclinD1	F: 5′-ACCCTGACACCAATCTCCTCAAC -3′ R: 5′-ATGGATGGCACAATCTCCCTCTGC-3′	118	56	NM_171992
p27^kip1^	F: 5′-TGTAGTGTCCTTTCGGTGAGAACTG-3′ R: 5′-GAATCTTCGGAACTCCCAAATGAG-3′	126	50	NM_031762.3
p27^kip1^	F: 5′-GACTTTCAGAATCATAAGCCCCTG -3′ R: 5′-TGGACACTGCTCCGCTAACC-3′	261	58	NM_009875
Hes5	F: 5′-GCTCAGTCCCAAGGAGAAAAATC -3′ R: 5′-GCTTTGCTGTGCTTCAGGTAGC-3′	192	58	NM_024383
Hes1	F: 5′-TTTGCCTTTCTCATCCCCAAC -3′ R: 5′-CCAAGTTCGTTTTTAGTGTCCGTC-3′	224	58	NM_024360
Lef1	F: 5′-CCCAGAAGGAGAAGATTTTCGC -3′ R: 5′-ACTGTGTTTGTCCGACCACCTC-3′	138	56	AF198533
Abcg 2	F: 5′-TCAGTTTATCCGTGGCATCTCTG -3′ R: 5′-GTTGTAGGGCTCACAGTGGTAACC-3′	325	56	NM_011920
Sox 2	F: 5′-AGGGCTGGGAGAAAGAAGAG -3′ R: 5′-GGAGAATAGTTGGGGGGAAG-3′	177	56	NM_011443
Mash1	F: 5′- CAACCAAATAATCCCAGAAGCAGG -3′ R: 5′- AAAGCAGCCGCAAAAGTCAGTGCC-3′	128	58	NM_008553.4
Rx	F: 5′-ATCCCAAGGAGCAAGGAGAG -3′ R: 5′-TTCTGGAACCACACCTGGAC-3′	256	58	AF135839
Nestin	F: 5′-TGGAGCAGGAGAAGCAAGGTCTAC -3′ R: 5′-TCAAGGGTATTAGGCAAGGGGG-3′	295	56	NM012987
GS	F: 5′-TCACAGGGACAAATGCCGAG -3′ R: 5′-GTTGATGTTGGAGGTTTCGTGG-3′	362	58	NM_008131
Vimentin	F: 5′-AAGGCACTAATGAGTCCCTGGAG -3′ R: 5′-GTTTGGAAGAGGCAGAGAAATCC-3′	251	56	NM_011701
Brn3b	F: 5′-GGCTGGAGGAAGCAGAGAAATC -3′ R: 5′-TTGGCTGGATGGCGAAGTAG-3′	141	58	NM_138944
Opsin	F: 5′-GGATGAGGGAGGAATGAGTGAC -3′ R: 5′-CACACACATACACACATCACACGC-3′	321	60	NM_22180
RK	F: 5′-GCTGAACAAGAAGCGGCTGAAG -3′ R: 5′-TGCTGTGTAGTAGATGGCTCGTGG-3′	238	56	NM_011881
B-actin	F: 5′-GTGGGGCGCCCCAGGCACCA-3′ R: 5′-CTCCTTAATGTCACGCACGATTTC-3′	548	50	XM_037235
GAPDH	F: 5′-ACAGTCCATGCCATCACTGCC -3′ R: 5′-GCCTGCTTCACCACCTTCTTG-3′	266	60	NM_017008.3

### Immunofluorescence analysis

Detection of specific markers and BrdU was performed as previously described [26]. Briefly, paraformaldehyde-fixed explants and cells were incubated in 1xPBS containing 5% NGS and 0.2 or 0.4% Triton X-100 followed by an overnight incubation at 4°C with the antibodies described in Table 2. Cells and sections were examined for epifluorescence after incubation in anti–species-specific immunoglobulin G conjugated to cyanin 3 (Cy3), fluorescein isothiocyanate (FITC), or 7-amino-4-methylcoumarin-3-acetic acid (AMCA). Slides were mounted with VectaShield (Vector Laboratories, Burlingame, CA) and captured using a cooled CCD camera (Princeton Instruments, Trenton, NJ) and Openlab software (Improvision, Lexington, Mass., USA) or Zeiss 510 Meta Confocal Laser Scanning Microscope. Adobe Photoshop CS4 was used for image presentation. Manipulation of the images was restricted to threshold and brightness adjustments to the whole image. Controls for the experiments consisted of the omission of primary antibodies. No staining was observed in these cases.

**Table 2:** List of primary antibodies used.

Name	Species	Dilution	Company
BrdU	Rat	1∶200	Accurate (OBT0030)
GS	Mouse	1∶100	Sigma (WH0002752M1)
GS	Rabbit	1∶5000	Sigma (G2781)
Pax6	Rabbit	1∶100	Covance (PRB-278P)
Musashi	Rabbit	1∶100	Millipore (AB5977)
Ret-P1	Mouse	1∶100	Gift
Sox9	Rabbit	1∶200	Millipore (AB5535)
Rx	Rabbit	1∶100	Santa Cruz (sc-79031)
Ki67	Mouse	1∶50	BD (556003)
Cleaved Caspase3	Rabbit	1∶200	Cell Signaling (9664)

### Optokinetic Test

The optokinetic response was measured using the OptoMotry System (CerebralMechanics, Lethbridge, AB, Canada). The testing arena was composed of a platform positioned 13 cm above the floor and four 20″ LCD computer monitors (model 2007FPb; Dell) attached to each outside wall, arranged in a square formation and projecting a three-dimensional (3-D) virtual cylinder comprising a vertical sine wave grating in coordinate space controlled by OptoMotry software. The software controlled the geometry of the cylinder and the speed and spatial frequency of the stimuli. A video camera (DCR-HC28; Sony) was positioned directly above the platform enabling a live video feedback of the testing arena. Animals were positioned unrestrained on the centered platform to allow the experimenter to follow the head's reflexive movements and eye's tracking in concert with the image rotation. After a brief adaptation period (with no projecting image), the observer assessed whether the animals tracked the cylinder by monitoring the image of the cylinder and the animal in the video window. If the rat's head tracked cylinder rotation, it was judged as a positive response, where the animal could see the grating. The analysis was restricted to the combined response obtained from each animal tested.

### Lineage tracing

#### *Lentivirus production and infection*

Lentivirus production was performed as previously described [23]. Briefly, lentivirus was packaged by transfecting 293T cells with Lipofectamine 2000 (in IMDM, 10% FBS, and 2 mM L-glutamine) using a plasmid system consisting of the pFTM3GW vector. The cells were plated the previous day in three T-75 flasks (coated with poly-L-lysine) at a density of 1.5×10^7^ cells/plate. Twelve hours after transfection, media with antibiotics (Penicillin (100 U/ml) and Streptomycin (100 µg/ml; Invitrogen) replaced the media containing the transfection reagents. Supernatants (containing the viral particles) were harvested 24 and 48 h after the first media change, and filtered through a 0.45 µm pore PVDF Durapore filter (Millipore Corp., Billerica, MA). The filtrates were centrifuged in 4 ultracentrifuge tubes (38.5 ml; number 344058; Beckman-Coulter) underlaid with 4 ml of sucrose (20%) for 2 h at 4°C. The pellets were resuspend in 800 µl cold PBS. After 30 min incubation on ice, the samples were pooled and centrifuged for 1.5 h at 4°C. The pellet was carefully resuspend in 120 µl cold PBS. After incubation on ice for 2 h, the virus was divided into 10 µl aliquots, and used immediately or stored at -80°C. The amount of 5 ul of GS lentivirus (about 5×10^11^ transducing units per ml) was added on the top of the explants (GCL) at the day 1 in culture. After 12 hours, the medium with the lentivirus was removed and substituted for fresh medium.

### Statistical Analysis

To determine the percentage of specific cell types in a particular condition, the number of BrdU^+^ and cell-specific antigen-positive cells were counted in 10-15 randomly selected fields in three to five different coverslips or 100 µm area in 5–10 retinal sections/treatment. Each experiment was repeated at least three times. Values were expressed as means/% ± s.e.m. Data was analyzed using the Student's *t*-test to determine the significance of the differences between treatment and control in various conditions. Q-PCR results were given in fold change, based on the individual gene expression modifications in comparison to the respective control of the experimental group. To determine the improvement in light perception, treated and control S334ter rats were subjected to optokinetic tests in two batches, consisting of 28 animals in the first batch and 31 in the second. Distributions of the control and treatment group measures were examined using boxplots and descriptive statistics. Wilcoxon ranksum tests were used to determine the statistical difference between the two groups. Spearman and Pearson correlation was used to determine the relationship between BrdU^+^GS^+^opsin^+^ cell number and visual function.

## Results

### Activation of Müller cells by Notch and Wnt signaling

**Figure 1:**
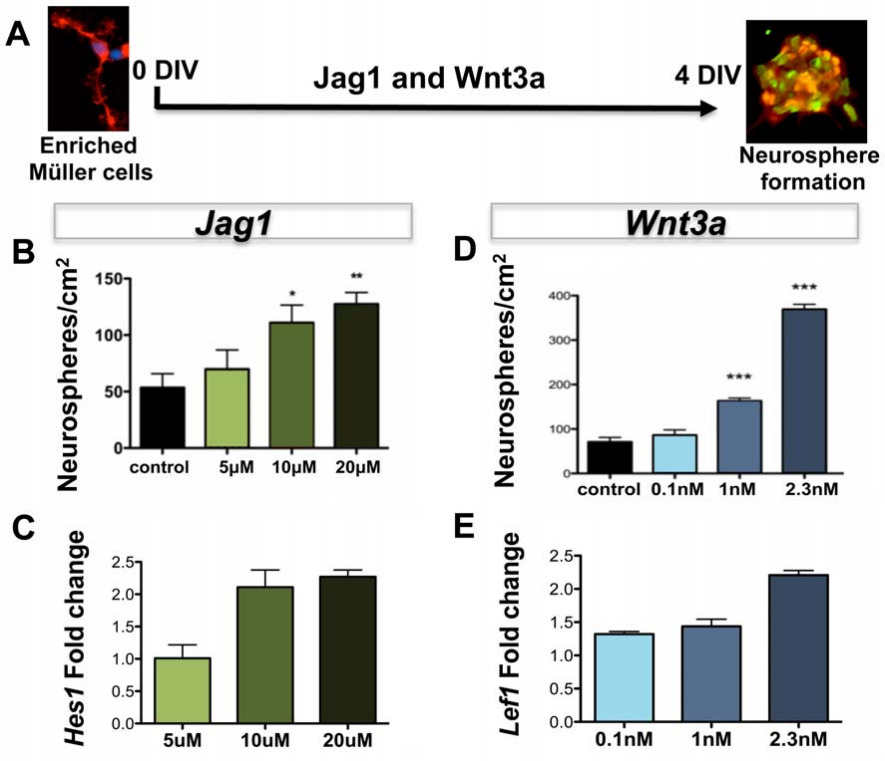
Effects of Notch/Wnt signaling on the activation of enriched Müller cells. (**A**) Schematic representation of a protocol for examining the effects of Notch and Wnt signaling on the activation of enriched Müller cells by a neurosphere assay (DAPI in blue, GS in red, and BrdU in green). There was a dose-dependent generation of neurospheres (**B**) and an accompanying increase in levels of *Hes1* transcripts (**C**) when cultured in the presence of Jag1, compared to controls. Similarly, there was a dose-dependent generation of neurospheres (**D**) and an accompanying increase in levels of *Lef1* transcripts (**E**) when enriched Müller cells were cultured in the presence of Wnt3a, compared to controls. * = p<0.01, ** = p<0.001, *** = p<0.0001.

**Figure 2:**
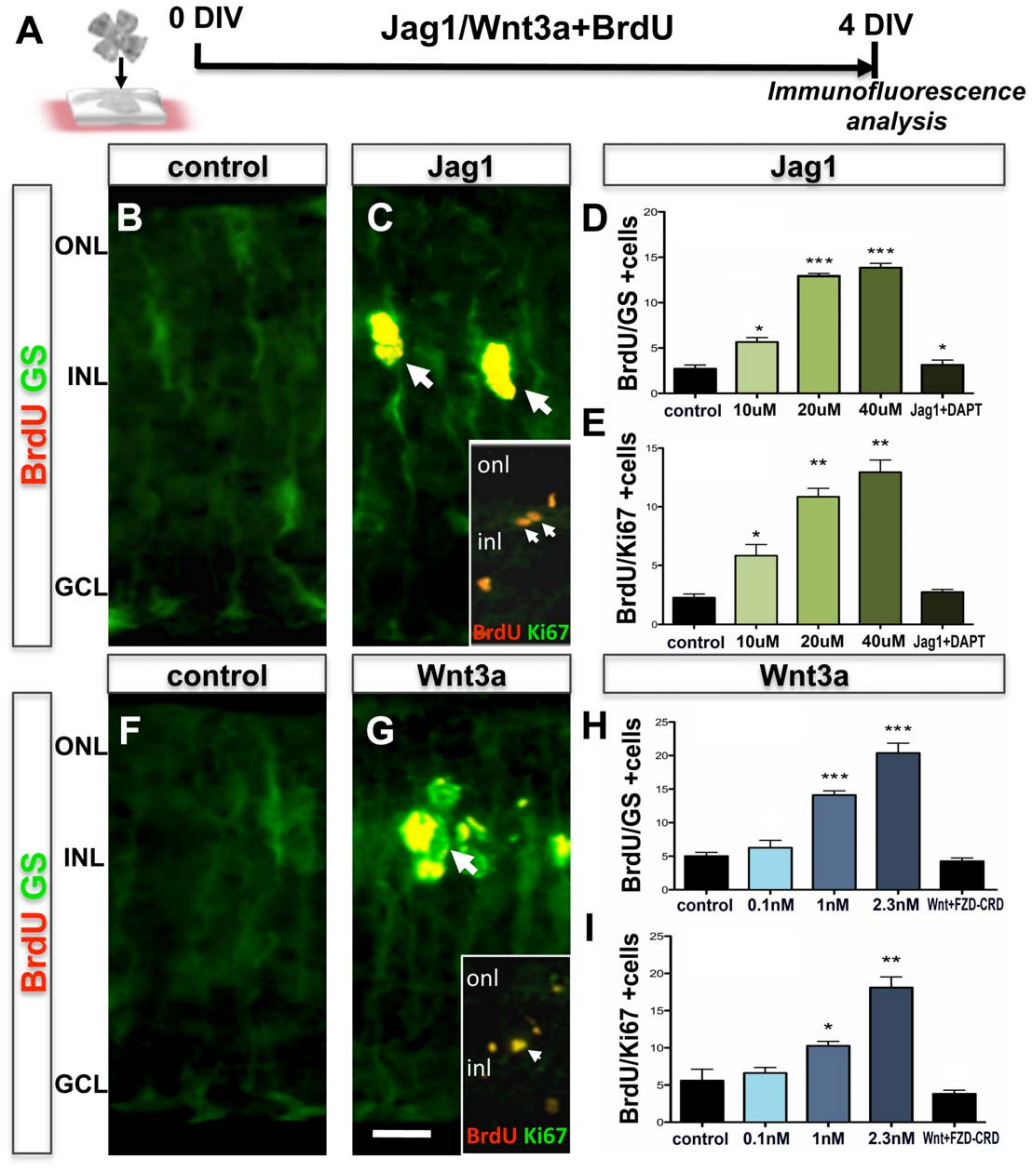
Effects of Notch/Wnt signaling on the activation of Müller cells in retinal explants. Schematic representation of a protocol for examining the effects of Notch and Wnt signaling on the activation of Müller cells in retinal explants from wild type mouse (PN21) by BrdU-incorporation (**A**). Immunofluorescence analysis of retinal explants cultured in the presence of Jag1 revealed BrdU^+^ cells co-expressing GS and Ki67 (inset) (arrows) (**B**, **C**) and the proportion of BrdU^+^GS^+^ (**D**) and BrdU^+^Ki67^+^ (**E**) cells increased in a dose-dependent manner, compared to controls. The effects were abrogated in the presence of DAPT. Similarly, immunofluorescence analysis of retinal explants cultured in the presence of Wnt3a revealed BrdU^+^ cells co-expressing GS and Ki67 (inset) (**F**, **G**) and the proportion of BrdU^+^GS^+^ (**H**) and BrdU^+^Ki67^+^ (**I**) cells increased in a dose-dependent manner, compared to controls. The effects were abrogated in the presence of FzdCRD. ONL = outer nuclear layer; INL = inner nuclear layer; GCL = ganglion cell layer. Scale  = 10 µm. * = p<0.01, ** = p<0.001, *** = p<0.0001.

We began by determining the specificity of the canonical Notch and Wnt pathways in activating Müller cells with dormant stem cell potential. First, the effects of the pathways were examined in enriched Müller cells culture, which is independent of any other cell type, and second, in cultured retinal explants, where retinal architecture and cell-cell interactions are maintained. In the former, the index of activation was the generation of clonal neurospheres and, in the latter, it was the number of GS-positive cells that had incorporated BrdU (BrdU^+^GS^+^ phenotype). To perturb Notch and Wnt signaling, a 17 amino acid peptide sequence corresponding to the DSL domain of Jagged1 peptide (Jag1) [27] and Wnt3a [13], respectively, were used (**Fig. 1A**). We observed a dose-dependent increase in the number of neurospheres when enriched Müller cells were cultured in the presence of Jag1 and Wnt3A, compared to controls (**Fig. 1B, 1D**). There was a 2.3 fold (p<0.001) and 5.2 fold (p<0.0001) increase in the number of neurospheres at the highest concentration of Jag1 (20 µM) and Wnt3a (2.3 nM), respectively. That the generation of neurospheres involved the canonical pathways was demonstrated by an accompanying dose-dependent increase in the levels of transcripts corresponding to *Hes1* and *Lef1*, which encode transducers of the canonical Notch and Wnt signaling pathways, respectively (**Fig. 1C, 1E**). Similarly, we observed a dose-dependent increase in the number of GS^+^BrdU^+^ cells in the sections of retinal explants treated with Jag1 (**Fig. 2B–D**) and Wnt3A (**Fig. 2F–H**). GS^+^BrdU^+^ cells were observed in the inner nuclear layer where Müller cells somas are located and there was a 1.76 fold (p<0.0001) and 2.9 fold (p<0.0001) increase in their number at the highest concentration of Jag1 (40 µM) and Wnt3a (2.3 nM), respectively, compared to controls. To rule out the possibility that BrdU^+^ cells were apoptotic, we examined whether or not these cells also expressed Ki67, a nuclear protein expressed by cells that are active in the cell cycle. We observed that Ki67 immunoreactivities were co-localized in BrdU^+^ cells and there was a dose-dependent increase in the number of BrdU^+^Ki67^+^ cells similar to that observed for GS^+^BrdU^+^ cells (**Fig. 2E, 2I**). The effects of Jag1 and Wnt3A on the increase in GS^+^BrdU^+^/BrdU^+^Ki67^+^ cells were abrogated in the presence of DAPT (**Fig. 2D, 2E**), an inhibitor of gamma secretase, an enzyme essential for the cleavage of Notch receptor to initiate intracellular signaling, and FzdCRD (**Fig. 2H, 2I**), a soluble FZD receptor that neutralizes Wnt ligands by binding them [13], [24], suggesting the involvement Notch and Wnt pathways in the activation of Müller cells.

**Figure 3:**
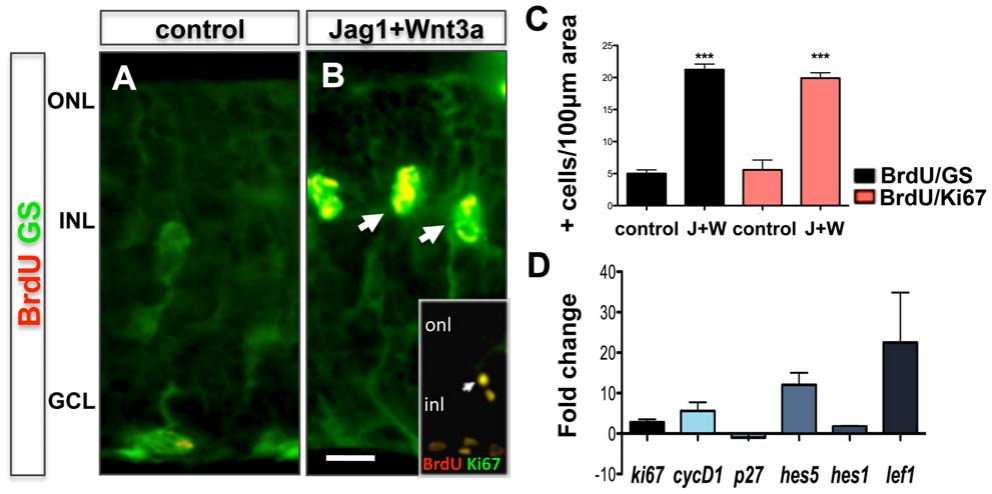
Müller cell activation in retinal explants by Notch and Wnt signaling. Immunofluorescence analysis of retinal explants cultured in the presence of Jag1 (20 µM)+Wnt3a (2.3 nM) revealed BrdU^+^ cells co-expressing GS and Ki67 (inset) immunoreactivities (**arrows**) (**A**, **B**) and the proportion of BrdU^+^GS^+^ and BrdU^+^Ki67^+^ increased compared to controls (**C**). Q-PCR analysis of gene expression revealed increase in levels of transcripts corresponding to *Ki67*, *cyclinD1*, *Hes1*, *Hes5* and decrease in *p27^kip1^* transcript levels in Jag1+Wnt3a treated explants, compared to controls (**D**). ONL = outer nuclear layer; INL = inner nuclear layer; GCL = ganglion cell layer. Scale  = 10 µm. *** = p<0.0001.

**Figure 4:**
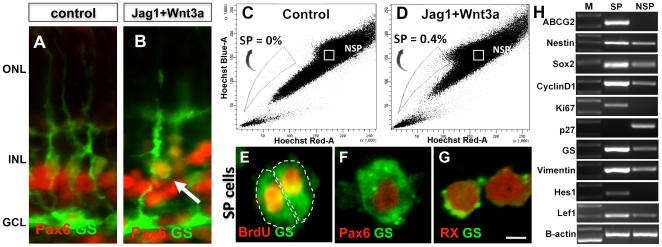
Neural properties of Notch and Wnt signaling activated Müller cells. Immunofluorescence analysis of retinal explants cultured in the presence of Jag1 (20 µM)+Wnt3a (2.3 nM) revealed GS^+^ cells expressing Pax6 immunoreactivities (arrow) (**B**) whereas such cells were absent in controls (**A**). Hoechst dye efflux assay revealed SP cells in Jag1+Wnt3a treated retinal explants (**D**) and in controls (**C**). Immunofluorescence analysis of SP cells revealed GS^+^ cells co-expressing immunoreactivities corresponding to BrdU (**E**), Pax6 (**F**), and Rx (**G**). RT-PCR analysis of SP and NSP cells from Jag1+Wnt3a treated retinal explants revealed a differential gene expression with transcripts corresponding to stem cell marker (*Abcg2*), neural progenitor markers (*Nestin* and *Sox2*), cell cycle regulators (*CyclinD1* and *Ki67*), and transducers of Notch (*Hes1*) and Wnt (*Lef1*) enriched in SP cells (**H**). In contrast, *p27^kip1^* transcripts were enriched in NSP cells. ONL = outer nuclear layer; INL = inner nuclear layer; GCL = ganglion cell layer, M =  Marker, SP =  Side Population, NSP = Non-Side Population. Scale  = 2.5 µm.

**Figure 5:**
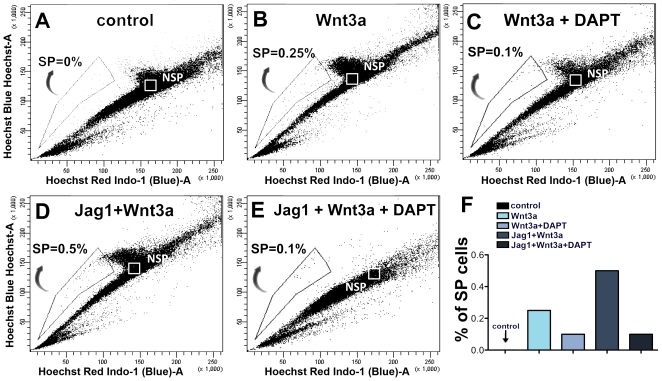
Interactions between Notch and Wnt signaling during the activation of Müller cells. Hoechst dye efflux assay revealed 0.25% of SP cells in total cell dissociates of retinal explants treated with Wnt3a (**B**), compared to controls (**A**). The proportion of SP cells decreased to 0.1% in retinal explants treated with Wnt3a+DAPT (**C**). The proportion of SP cells increased to 0.5% in retinal explants treated with Jag1+Wnt3a (**D**), which was decreased to 0.1% (**E**) when DAPT was added to the culture, suggesting a synergistic interaction between Notch and Wnt signaling in the activation of SP cell phenotype in Müller cells (**F**).

Next, the neurogenic potential of Müller cells was examined using retinal explants that were cultured in the presence Jag1 (20 µM) + Wnt3a (2.3 nM) + BrdU (50 µM) for 4 days (activation phase). Results from these experiments demonstrated a significant increase (3.24 fold, p<0.0001) in the number of GS^+^BrdU^+^ cells in the inner nuclear layer of explant sections, compared to controls (**Fig. 3A–C**). However, compared to the effects of Jag1 and Wnt3A alone, we did not observe a synergistic effect of Jag1+Wnt3A treatment on the number of GS^+^BrdU^+^ cells. To understand the underlying mechanism by which Jag1+Wnt3a activated Müller cells, we examined the expression of transcripts corresponding to cyclin-dependent kinase (CDK) activator (CyclinD1), inhibitor (p27^kip1^), and marker of the active phases of the cell cycle (Ki67) (**Fig. 3D**). We observed an increase in levels of *CyclinD1* and *Ki67* transcripts and a decrease in *p27^kip1^* transcript levels, suggesting that an accentuation of CDK activities underlie Jag1+Wnt3A-mediated activation of Müller cells. That these effects involved the canonical Notch and Wnt pathways was demonstrated by an increase in levels of *Hes1/Hes5* and *Lef1* transcripts, respectively. Next, whether or not the activated Müller cells possessed the phenotype of neural progenitors was investigated (**Fig. 4**). Immunohistochemical analysis of Jag1+Wnt3A-treated retinal explants revealed GS^+^ cells in the inner nuclear layer, co-expressing immunoreactivities corresponding to Pax6. Since GS^+^Pax6^+^ cells were absent in the control retinal explants, this suggests that some Müller cells in Jag1+Wnt3A-treated retina acquired the expression of the neural marker Pax6 (**Fig. 4A** and **4B**). For an unambiguous determination of neural phenotype of the activated Müller cells, cells were dissociated from treated and control retinas and subjected to a Hoechst dye efflux assay, which enriched activated Müller cells as Side Population (SP) cells [13]. The SP phenotype is a universal feature of stem cells, conferred by the expression of Abcg2, an ATP-binding cassette (ABC) transporter [5], [26], [28], [29], [30]. We observed approximately 0.4% of the cells in the Jag1+Wnt3A-treated retinal explants were SP cells, whereas none were detected in controls (**Fig. 4C ** and ** 4D**). The SP is heterogeneous; the lower SP compartment, near the tip of the gate, contains cells that are relatively mitotically quiescent, and the upper compartment toward the NSP, contains rapidly proliferating cells [31], [32], [33]. Therefore, SP cells from Jag1 and Wnt3A samples were preferentially localized in the upper compartment (**Fig.** **4D** **)**. The SP cells were GS^+^, affirming their Müller cell lineage, and the majority of them were BrdU^+^, confirming their proliferative status (**Fig. 4E**). The SP cells expressed immunoreactivities corresponding to neural progenitor markers, Pax6 and Rx (**Fig. 4F, 4G**). Further examination of the phenotype by RT-PCR analysis revealed that SP cells were enriched in transcripts corresponding to general stem cell marker (*Abcg2*), neural progenitor markers (*Nestin and Sox2*), positive regulators and a marker of the cell cycle (*CyclinD1* and *Ki67*), Müller cell markers (*GS* and *Vimentin*), and transducers of Notch (*Hes1*) and Wnt (*Lef1*) signaling (**Fig. 4H**). Transcripts corresponding to CDK inhibitor *p27^kip1^* were nearly absent in SP cells. These observations confirmed the proliferative nature and neural phenotype of the activated Müller cells. Next, we examined whether or not the emergence of the Müller SP cell phenotype was due to the interactions between Wnt and Notch signaling as observed in the case of retinal progenitors during retinal histogenesis [34]. There was a 2-fold increase in the number of Müller SP cells in Jag1+Wnt3a-treated retinal explants compared to those cultured in the presence of Wnt3a (**Fig. 5B, 5D**). The proportion of Müller SP cells was abrogated to 0.1% in the presence of DAPT in both treated groups (**Fig. 5C, 5E**). SP cells were not detected in control (**Fig. 5A**). These observations illustrate a synergism between Notch and Wnt signaling during the activation of Müller cells, where the former sets the threshold for the influence of the latter.

### Differentiation of activated Müller cells along rod photoreceptor lineage in wild type mice retina *in vitro*

**Figure 6:**
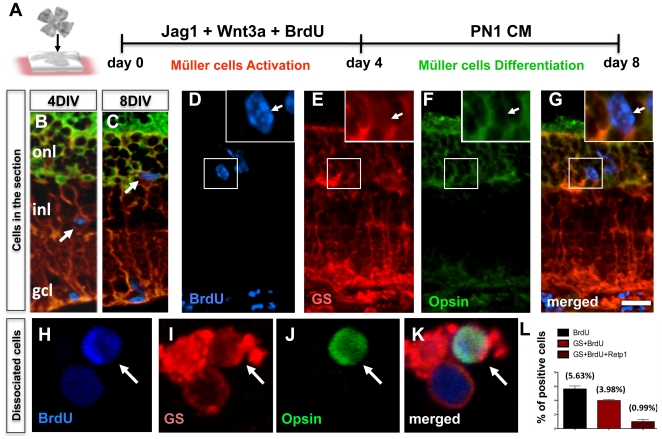
Differentiation of activated Müller cells along rod photoreceptor lineage in wild type mice retina *in vitro.* Schematic representation of a protocol for examining the neurogenic potential of Notch + Wnt signaling activated Müller cells (**A**). Immunofluorescence analysis of Jag1+Wnt3a treated retinal explants at 4DIV and 8DIV (days in vitro), demonstrates BrdU^+^ cells preferentially localized in the INL (**B**) and ONL (**C**) (arrows), respectively. BrdU^+^ cells in the ONL at 8DIV co-expressed immunoreactivities corresponding to GS and opsin (D–G) (arrows). Immunofluorescence analysis of cell dissociates from retinal explants at 8DIV revealed BrdU^+^ cells expressing GS and opsin immunoreactivities (**H–K**). The proportion of BrdU^+^, BrdU^+^GS^+^ and BrdU^+^GS^+^opsin^+^ cells is given in **L**. ONL = outer nuclear layer; INL = inner nuclear layer; GCL = ganglion cell layer. Scale  = 20 µm.

**Figure 7:**
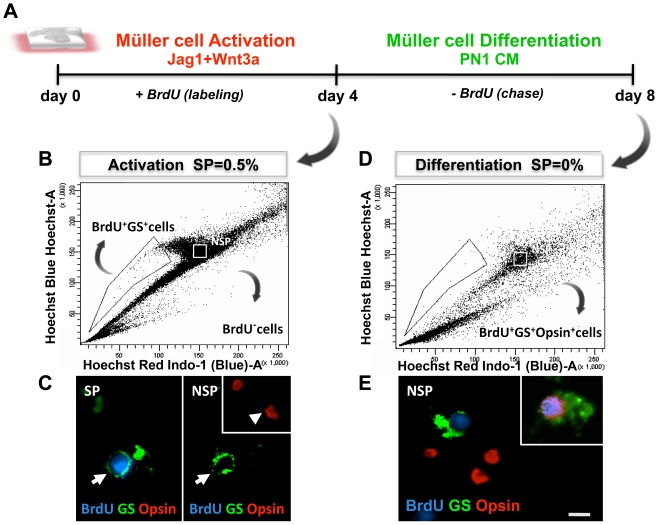
Differentiation of Müller SP cells along rod photoreceptor lineage. Schematic representation of the activation of Müller SP cells and the chase of their fates at the end of the differentiation phase (**A**). Hoechst dye efflux analysis of Jag1+Wnt3a treated retinal explants at the end of the activated phase (4DIV) revealed 0.5% of SP cells in cell dissociates (**B**). SP cells were BrdU^+^GS^+^ and opsin^−^ (arrow) whereas NSP cells were all BrdU^−^ and some were GS^+^ (arrows) and some opsin^+^ (arrowhead) (**C**). Hoechst dye efflux analysis of Jag1+Wnt3a treated retinal explants at the end of the differentiation phase (8DIV) revealed the absence of SP cells (**D**). Immunofluorescence analysis of NSP cells revealed BrdU^+^ GS^+^ cells, some of which co-expressed opsin immunoreactivities, suggesting that BrdU^+^ SP cells lost their SP cell phenotype and shifted to NSP where they differentiated along rod photoreceptor lineage (**E**). Scale  = 5 µm.

**Figure 8:**
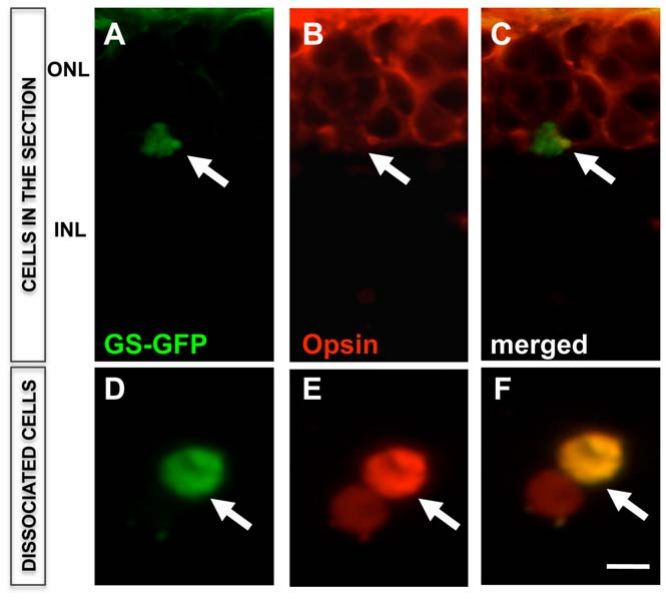
Lineage tracing of activated Müller cell differentiation along rod photoreceptor lineage. GS-GFP lentivirus transduced retinal explants from wild type mice (PN21) were cultured in the presence of Jag1+Wnt3a for 4 days followed by differentiation for 4 days in the presence of PN1 rat retinal conditioned medium. Immunofluorescence analysis of retinal explant sections (**A–C, arrows**) and cell dissociates (**D–F**) at the end of the differentiation phase (8DIV) revealed GFP expressing Müller cells co-expressing opsin. ONL = outer nuclear layer; INL = inner nuclear layer. Scale  = 2.5 µm.

**Figure 9:**
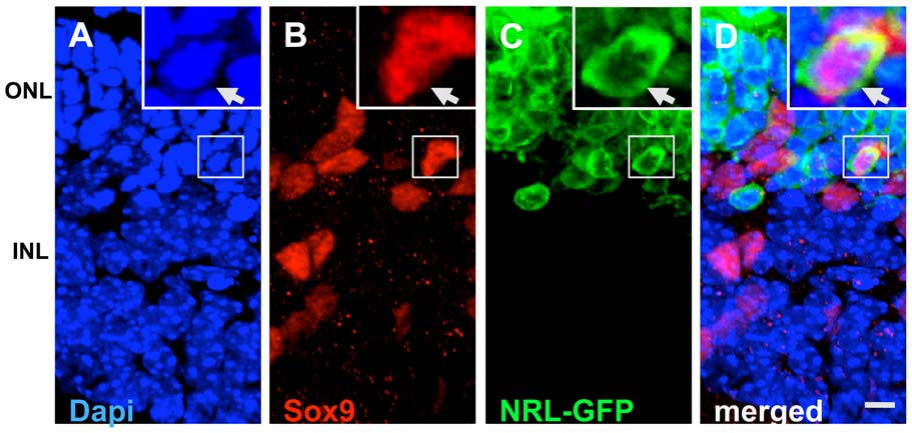
Genetic evidence of activated Müller cell differentiation along rod photoreceptor lineage. Retinal explants from Nrl-GFP mice (PN21) were cultured in the presence of Jag1+Wnt3a for 4 days followed by differentiation for 4 days in the presence of PN1 rat retinal conditioned medium. Immunofluorescence analysis of retinal explant sections at the end of the differentiation phase revealed Sox9 expressing Müller cells co-expressing GFP under the influence of rod photoreceptor regulator, Nrl (A–D, arrows). ONL = outer nuclear layer; INL = inner nuclear layer. Scale  = 10 µm.

To examine the regenerative potential of the activated Müller cells, retinal explants were examined at the end of 4 days of culture in the presence of PN1 retinal conditioned medium (PN1CM). PN1CM was used, as these cells have been observed to elaborate potent rod photoreceptor-promoting activities, capable of inducing retinal progenitors [35], [36], embryonic stem (ES) cells [37], limbal stem cell-derived neural progenitors [38], and iPS cells [39] along rod photoreceptor lineage (**Fig. 6A**). Examination of the spatial location of GS^+^BrdU^+^ cells at the end of the activation and differentiation phases showed that these cells were preferentially located in the INL and ONL, respectively, suggesting a migration of activated Müller cells (GS^+^BrdU^+^ cells) to the ONL in differentiation conditions (**Fig. 6B, 6C** **)**. A rare subset of GS^+^BrdU^+^ cells was detected in the outer nuclear layer of the retina expressing immunoreactivities corresponding to opsin, suggesting differentiation along rod photoreceptor lineage (**Fig. 6D–G**). To confirm the BrdU^+^GS^+^opsin^+^ phenotype unambiguously and estimate the proportion of such cells, retinal explants at the end of the differentiation phase were dissociated and subjected to triple immunofluorescence analysis. Cells with the BrdU^+^GS^+^opsin^+^ phenotype could be identified unambiguously (**Fig. 6H–K** **)** and their proportion in total cells was less than 1% (0.99%, p<0.0001) (**Fig. 6L**). The specificity of the differentiation of activated Müller cells along the rod photoreceptor lineage was determined as follows: First, we examined the fate of Müller SP cells as they shifted from the SP cell compartment during the activation phase to the non-SP (NSP) compartments during the differentiation phase (**Fig. 7A**). The logic behind this experiment is that Müller SP cells, upon differentiation, would down-regulate the expression of *Abcg2* and lose their SP phenotype. Thus, these cells, which were tagged with BrdU during activation, instead of segregating out in SP compartment, will be detected in NSP compartment upon differentiation. As expected, we observed that at the end of the activation phase, GS^+^BrdU^+^cells were exclusively localized in the SP compartment and absent from the NSP compartment (**Fig. 7B, 7C**). At the end of the differentiation phase, the SP compartment was found depleted of cells and GS^+^BrdU^+^ cells were now observed in the NSP compartment (**Fig. 7E**), which contains post-mitotic precursors and differentiated cells. A small subset of GS^+^BrdU^+^ cells in the NSP compartment expressed immunoreactivities corresponding to opsin (**Fig. 7E** **, inset**), suggesting that activated Müller SP cells lost their SP phenotype upon differentiation and shifted to NSP compartment, where some of them differentiated along the rod photoreceptor lineage. Second, we cultured retinal explants during the activation phase in the presence of GS-GFP lentivirus [23] for lineage tracing of the activated Müller cells. Cells with a GFP^+^opsin^+^ phenotype were detected in both sections (**Fig. 8A–C**) and dissociated cells (**Fig. 8D–F**) of the retinal explants, thus demonstrating that Müller cells, which were capable of activating GS promoter acquired rod photoreceptor phenotype. Lastly, to obtain genetic evidence for Müller cell-mediated photoreceptor regeneration, we cultured retinal explants from PN21 Nrl-GFP mice in the presence of Jag1+Wnt3A as previously described. In these animals, rod photoreceptors are identified by the expression of GFP driven by promoter activities of *Nrl* [22]. We observed a minor population (0.91%±0.47) of GFP-positive cells expressing Sox9, a homeodomain transcription factor characteristic of Müller cells [40], suggesting that activated Müller cells have been reprogrammed to activate promoter of *Nrl*, the transcriptional regulator of *Opsin* gene (**Fig. 9A–D**). Together these observations suggested that a rare subset of Müller cells activated by Notch and Wnt signaling differentiate along the rod photoreceptor lineage in the explants of wild type retinal explants.

### Differentiation of activated Müller cells along rod photoreceptor lineage in *rd* mice retina *in vitro* and in S334ter rat retina *in vivo*

**Figure 10:**
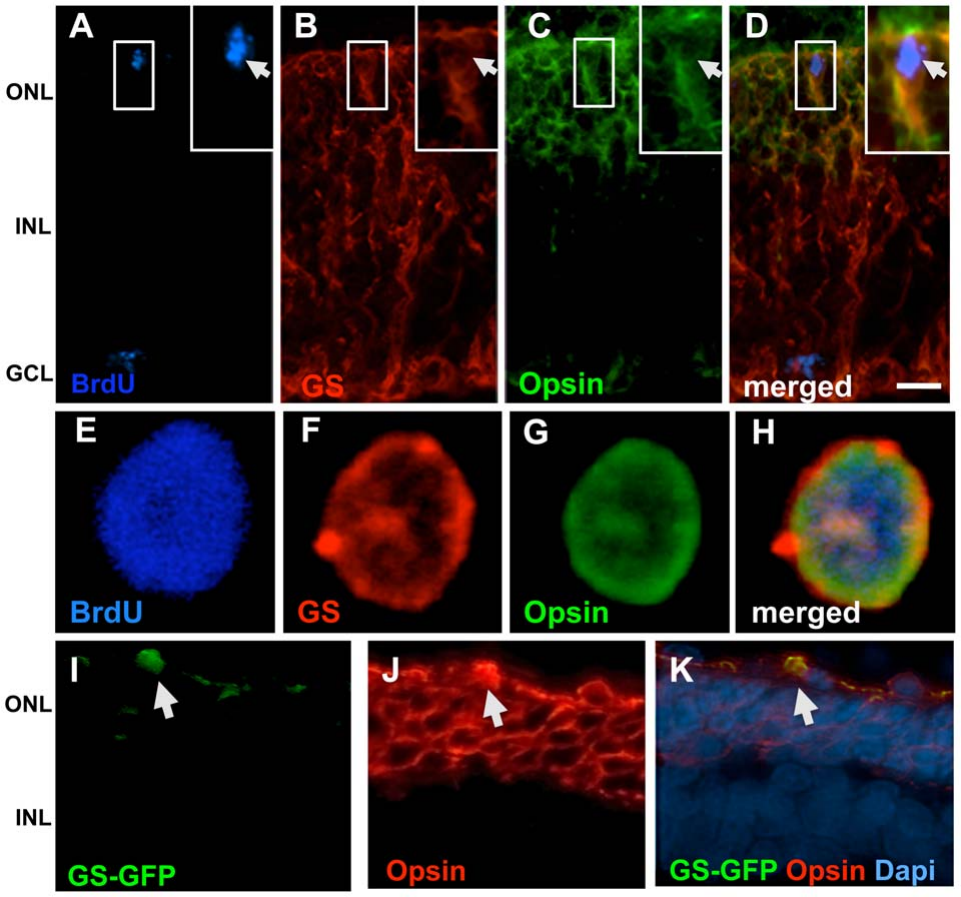
Rod photoreceptor differentiation of activated Müller cells in *rd* mice *in vitro*. Retinal explants from *rd* mice (PN10) were cultured in the presence of Jag1+Wnt3a for 4 days followed by differentiation for 4 days in the presence of PN1 rat retinal conditioned medium. Immunofluorescence analysis of retinal explant sections (**A–D**, arrows) and cell dissociates (**E–H**) at the end of the differentiation phase (8DIV) revealed BrdU^+^GS^+^ Müller cells co-expressing opsin. Immunofluorescence analysis of sections of retinal explants that were transduced with GS-GFP lentivirus and subjected to differentiation protocol as above revealed GFP expressing cells co-expressing opsin (**I-K**, arrows). ONL = outer nuclear layer; INL = inner nuclear layer; GCL = ganglion cell layer. Scale  = 20 µM.

**Figure 11:**
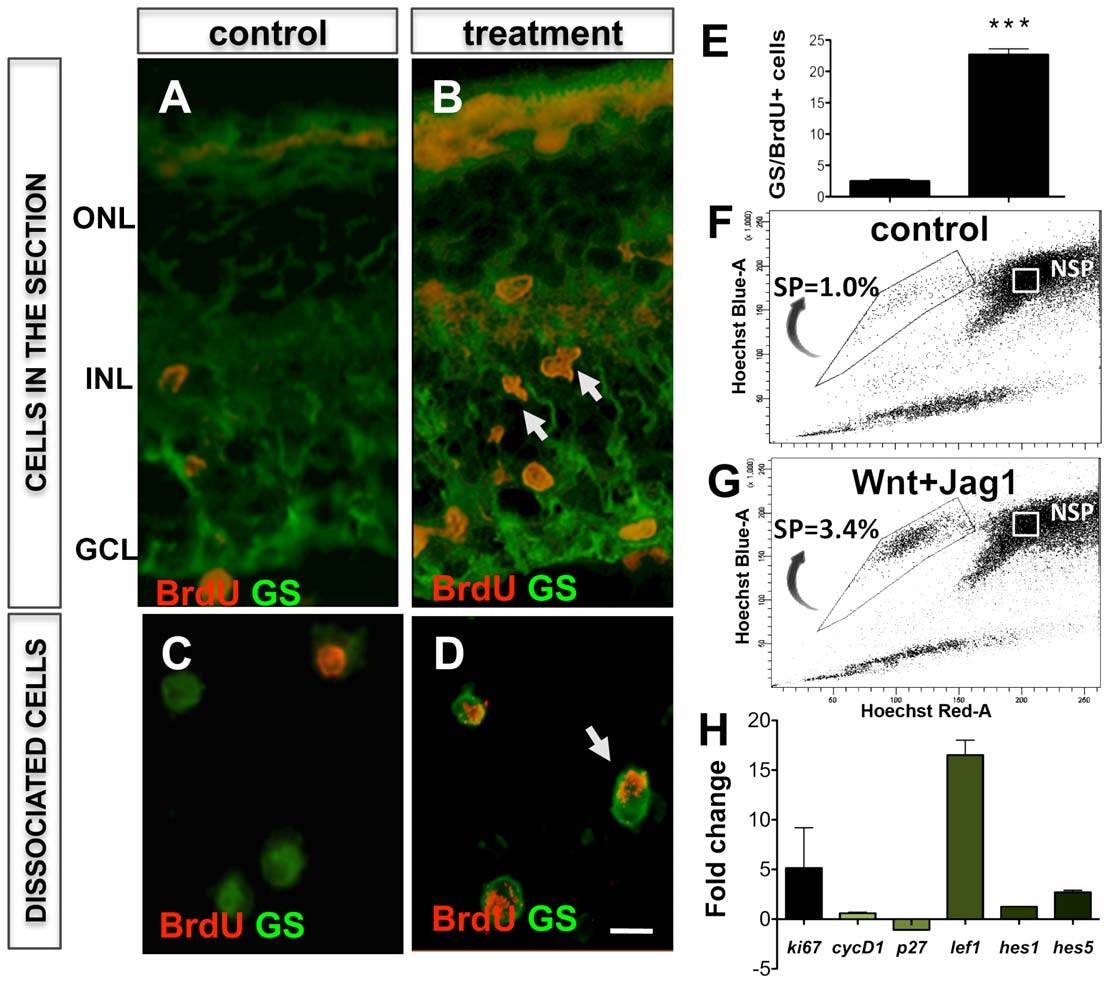
Notch and Wnt signaling-mediated activation of Müller cells in S334ter rats *in vivo*. S334ter rats received intravitreal injections of Jag1+Wnt2b at PN10 and PN11, followed by immunofluorescence, Hoechst dye efflux and Q-PCR analyses on PN 13. Immunofluorescence analysis of retinal sections (**A**, **B**, arrows) and cell dissociates revealed the presence of BrdU^+^ cells co-expressing GS (**C**, **D**), whose proportion was significantly higher than in controls (**E**). Hoechst dye efflux assay revealed a higher proportion of SP cells (3.4%) in Jag1+Wnt2b treated retina, compared to that in controls (1%) (**F**, **G**). Q-PCR analysis of gene expression revealed increase in levels of transcripts corresponding to *Ki67*, *cyclinD1*, *Hes1*, *Hes5* and decrease in *p27^kip1^* transcript levels in Jag1+Wnt2b treated retina, compared to controls (**H**). ONL = outer nuclear layer; INL = inner nuclear layer; GCL = ganglion cell layer. Scale  = 5 µM. *** = p<0.0001.

We next tested the premise of Müller cell-mediated photoreceptor regeneration in explants of *rd* mice where rod cells degenerate precipitously due to a mutation in the phosphodiesterase gene, using the activation-differentiation paradigm described previously. Similar to the wild type mice retinal explants, a significant increase in BrdU^+^GS^+^ cells was observed in Jag1+Wnt3a-treated *rd* mice retinal explants, compared to controls, accompanied by an increase in the expression of transcripts corresponding to *Ki67*, *CyclinD1*, *Hes1*, *Hes5*, and *Lef1*, and an increase in SP cells numbers with transcripts characteristic of proliferating neural progenitors (**Fig. S2**). Examination of explant sections at the end of the differentiation period revealed a subset (1.17%±0.46) of BrdU^+^GS^+^ cells in the degenerated outer nuclear layer expressing opsin immunoreactivities, thus suggesting their differentiation along the rod photoreceptor lineage (**Fig. 10A–D**). The BrdU^+^GS^+^opsin^+^ phenotype of differentiated cells was confirmed in cells dissociated from treated explants (**Fig. 10E–H**). Cells with the BrdU^+^GS^+^opsin^+^ phenotype were not detected in controls. The specificity of the differentiation of the activated Müller cells along the rod photoreceptor lineage was determined by lineage tracing; examination of retinal explants transduced with GS-GFP lentivirus at the end of the differentiation phase revealed GFP^+^ cells in the degenerated ONL expressing opsin immunoreactivities (**Fig. 10I–K**). Together, these observations suggest that the Notch and Wnt signaling-activated Müller cells in *rd* mice retina could differentiate along the rod photoreceptor lineage. Lastly, we tested the premise of Müller cell-mediated regeneration *in vivo* using S334ter rats, where a premature termination of opsin mRNA translation leads to rod photoreceptor degeneration [41]. Animals received intravitreal injection of a mixture of Jag1+Wnt2b+BrdU at PN10 to activate Müller cells at the onset of degeneration. Examination of retinal sections at the end of the activation phase revealed a 7.3 fold (p<0.0001) increase in the number of GS^+^BrdU^+^ cells in Jag1+Wnt2b-treated rats, compared to controls (**Fig. 11A–E**). Hoechst dye efflux assays revealed a ∼3-fold increase in Müller SP cell numbers in Jag1+Wnt2b-treated rats, compared to controls (**Fig. 11F, 11G**). The activation of Müller cells was accompanied by an increase in the levels of transcripts corresponding to *CyclinD1/Ki67* and a decrease in those corresponding to *p27^kip1^*, confirming the premise *in vivo* that an activation of *CyclinD1* and inhibition of *p27^Kip1^* expression underlie the G1-S transition in a subset of Müller cells' response to Notch and Wnt signaling. That these effects involve the canonical Notch and Wnt pathways was demonstrated by an increase in levels of *Hes1, Hes5* and *Lef1* transcripts, respectively (**Fig. 11H**).

**Figure 12:**
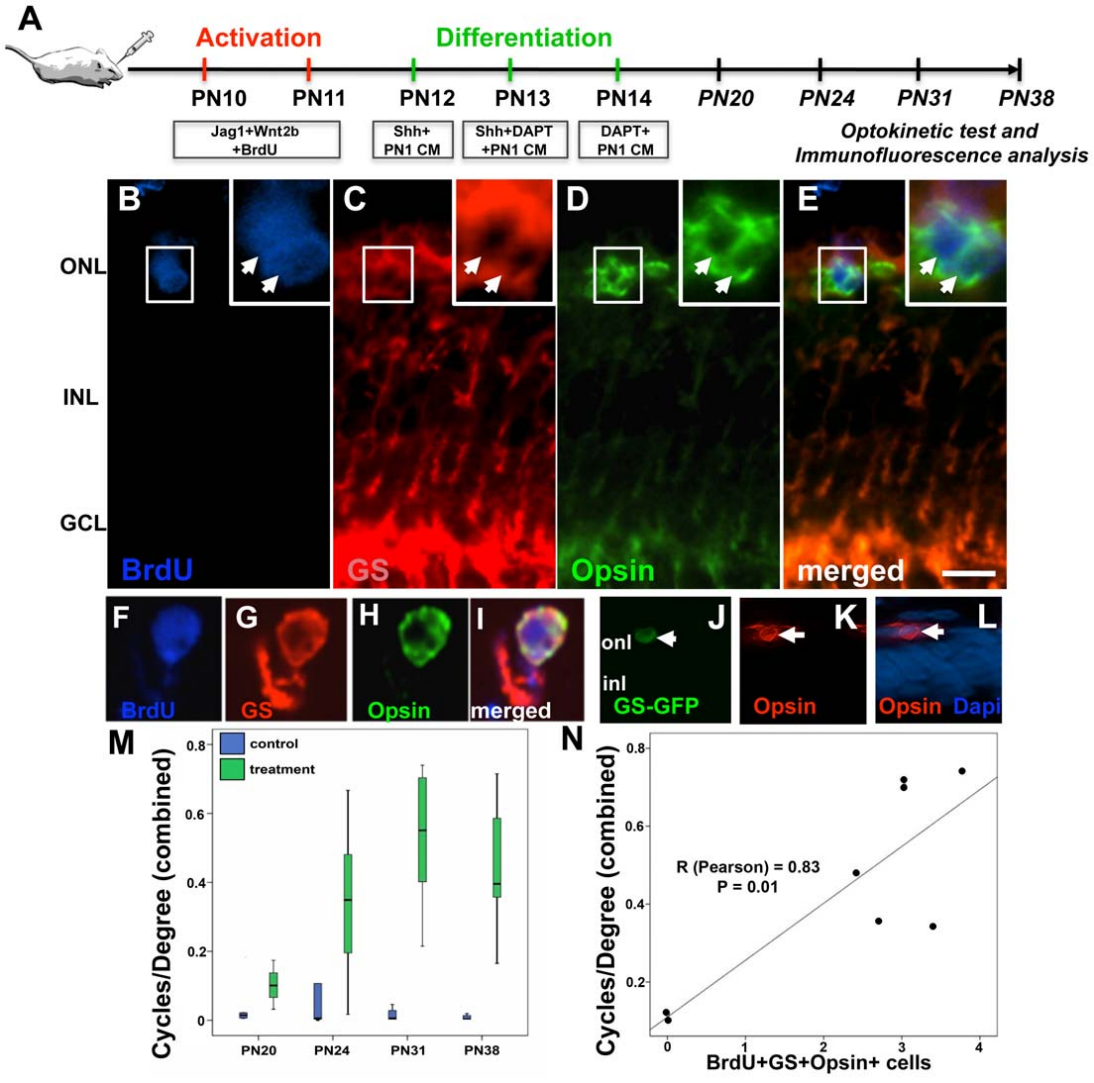
Differentiation of activated Müller cells along rod photoreceptor lineage in S334ter rats *in vivo*. Schematic representation of a protocol for examining the neurogenic potential of Notch + Wnt signaling activated Müller cells *in vivo* (**A**). Immunofluorescence analysis of retinal sections (**B–E**) and cell dissociates (**F–I**) at the differentiation phase (PN31) revealed BrdU^+^GS^+^ Müller cells co-expressing opsin (arrows). Immunofluorescence analysis of sections of retinal that were transduced with GS-GFP lentivirus and subjected to differentiation protocol as above, revealed GFP expressing cells co-expressing opsin (arrows) (**J–L**). Examination of the optokinetic response by boxplots revealed significant differences between treated and untreated groups at all stages post-treatment. The length of the box represents the interquartile range (the distance between the 25^th^ and 75^th^ percentiles) (**M**). The horizontal line in the box represents the median. The vertical lines represent the minimum and maximum values of the analysis variables. A correlation analysis between the proportions of BrdU^+^GS^+^opsin^+^ cells in cell dissociates of treated retina and optokinetic response (cycles/degree) of those animals shows a correlation coefficient of 0.83 and p = 0.01 (**N**). ONL = outer nuclear layer; INL = inner nuclear layer; GCL = ganglion cell layer. Scale  = 20 µM.

After 2 days of activation with Jag1 and Wnt2b, animals received intravitreal injections of Shh+PN1CM, followed by Shh+DAPT+PN1CM, and DAPT+PN1CM on consecutive days to promote differentiation (**Fig. 12A**). The treated and control (sham treated) retinas were subjected to immunohistochemical analyses following the optokinetic tests on animals at PN20, PN24, PN31 and PN38. As observed in the case of retinal explants, GS^+^BrdU^+^ cells were detected in the outer nuclear layer of the treated retina and a rare subset (2.1%±0.6) of these cells displayed the GS^+^BrdU^+^opsin^+^ phenotype (**Fig. 12B–E**). This phenotype was confirmed in cells dissociated from treated retina (**Fig. 12F–I**, **S3**). Such cells were rarely detectable either in sections or cell dissociates of control retina. In order to determine the specificity of Müller cell-based photoreceptor regeneration, a subset of animals received intravitreal injections of GS-GFP lentivirus during the activation phase. Examination of sections of lentivirus-transduced retina at PN31 revealed GFP^+^ cells expressing opsin immunoreactivities, confirming the differentiation of lineally tagged Müller cells along the rod photoreceptor lineage (**Fig. 12J–L**). To know whether Müller cell-derived opsin positive cells have functional implications, treated and control S334ter rats were subjected to an optokinetic test, a behavioral test of light perception measuring head and neck movement in response to a visual stimulus of rotating black and white stripes. The results of the optokinetic test revealed a significant temporal improvement in light perception, peaking at PN31, 17 days after the activation of Müller cells (**Fig. 12M**). Control rats, where the thickness of the ONL was reduced to one cell layer, did not display a significant optokinetic response. To establish a correlation between the observed improvement in light perception and the differentiation of Müller cells along the rod photoreceptor lineage, we quantified the cells with GS^+^BrdU^+^opsin^+^ phenotype in cell dissociates of the retina of the animals that had undergone optokinetic test. A correlation (R = 0.83; p = 0.01) was observed between the number of GS^+^BrdU^+^opsin^+^ cells and the corresponding optokinetic response (**Fig. 12N**). Together, these observations suggested that Müller cells could be activated in response to accentuation in Notch and Wnt signaling *in vivo* and a rare population of activated Müller cells differentiate along the rod photoreceptor lineage and the differentiation is correlated with the improvement in the perception of light in S334ter rat retina.

## Discussion

We have previously demonstrated that a subset of Müller cells enriched from adult mammalian retina, like their radial glia counterparts, retain the cardinal features of neural stem cells: self renewal potential and the ability to generate neurons and glia [13]. The enriched Müller cells generated generic neurons with biochemical, molecular, and physiological characteristics that had the capacity to differentiate into specific retinal neurons *in vitro*. As direct evidence of their neurogenic potential *in vivo*, we demonstrated that Müller cells, prospectively enriched as SP cells, integrated and generated lamina-specific retinal neurons after transplantation into mechanically injured retina [13]. These observations, including the more recent ones emerging from the lineage tracing experiments carried out in Zebra fish [14], [15], [16] and mice [21], show that the neurogenic potential of adult Müller cells is an inherent but dormant feature in many species. These cells express multiple stem cell/progenitor-specific genes whose expression is environment-sensitive [13]. The dual phenotype of Müller cells is reminiscent of radial glia and suggestive of an ambivalent nature that allows them to perform various roles that support neuronal functions and participate in neurogenesis, when the microenvironment allows. The mechanism that activates these dormant Müller cells is likely to involve pathways that integrate both cell-intrinsic and cell-extrinsic pathways and evidence suggests that Notch and Wnt signaling plays an important role in this regard [13]. A consistent feature of neurotoxin-induced activation of Müller cells is the increase in the expression of the Notch and Wnt pathways components. This suggests that injury induced cytokines and growth factors leading to the activation of Notch [42], [43] and Wnt [44], [45] pathways in Müller cells, prompts them to shift from maintenance to stem-cell mode. Our results demonstrate that the activation of Notch and Wnt signaling is sufficient to facilitate the re-entry of a subset of Müller cells into the cell cycle and does not require neurotoxin-mediated damage to the retina. Furthermore, these pathways act cooperatively toward the activation of Müller cells where Notch signaling calibrates the threshold of Wnt signaling; in the absence of Notch signaling, the effects of Wnt signaling are compromised as observed in the case of embryonic retinal stem cells/progenitors [34]. Based on previous observations that p27^kip1^ keeps Müller cells from entering the cell cycle [46] and the recent evidence that Notch and Wnt signaling adversely affect the activities of p27^kip1^ [47], [48], [49], it is reasonable to postulate that Notch and Wnt signaling-dependent inhibition of p27^kip1^ may constitute one of the first steps in the shift of Müller cells to the stem cell mode. The process is furthered by the expression of cell cycle regulators such as *cyclins*, promoters of stem cell homeostasis such as *Abcg2* [5], [28], and neurogenic genes such as *Sox2* [50], *Pax6* [51], [52], and *Rx* [53,].

The Notch and Wnt signaling activated Müller cells, albeit in an exceedingly low number, in three different animal models, display the ability to acquire rod photoreceptor phenotypes, suggesting that the neurogenic property of Müller cells that allows them to regenerate photoreceptors in lower vertebrates [14], [16] is evolutionary conserved. There are several interesting questions, directly related to the practical application of these observations, in treating retinal degeneration. First, why GS expression persisted in regenerated photoreceptors and what could be its functional implications? The reprogramming of Müller cells toward rod photoreceptors is likely to be a temporal process and the 2–3 weeks end points of our analysis may not be long enough to have erased the parental gene expression and hence, the co-localization of both GS and opsin in regenerated cells. While further studies, besides the indirect evidence (see below), are required to confirm whether or not such cells could be functional, trans-differentiated cells with residual parental properties have been observed to acquire the functions of converted lineage [54], [55], [56]. Second, why the event of neural conversion is so rare? There could be two possibilities, which are not mutually exclusive. The milieu of the adult mammalian retina is not conducive for regeneration and/or unlike Zebra fish Müller cells, their mammalian counterparts are intrinsically constrained to generate retinal neurons, hence the low efficiency of differentiation along neuronal lineages. Evidence favors first possibility for several reasons. First, there is a temporal change in the milieu from neonatal to adult retina, which leads to a progressive decline in the ability of the retina to support neurogenesis [53]. Second, the observation that a subset of Müller cells could engage Notch and Wnt pathways and activate the expression of genes corresponding to intrinsic regulators of the cell cycle [13], chromatin remodeling [57], and neurogenic potential [13] suggest that they are intrinsically capable of being neural stem cells. In light of this premise, the activated Müller cells reflect the behavior of embryonic retinal stem cells/progenitors which, when transplanted in the retina, differentiate into rod photoreceptors at a very low efficiency as opposed to when transplanted after commitment to the rod photoreceptor lineage where they not only express markers corresponding to rods, but also acquire morphological and functional differentiation [58]. Based on these observations, it can be surmised that the activated Müller cells possess the neurogenic capacity as seen in lower vertebrates, but unlike them they face an inhospitable environment for neuronal differentiation. This environment does not adequately support the progression of the activated Müller cells to rod precursors, the step essential for becoming rod photoreceptors. In our experiments, a minor population of Müller cell-derived rod photoreceptors was reproducibly observed when activated Müller cells were exposed to rod photoreceptor promoting conditions *in vitro* (**Fig. 6**) or *in vivo* (**Fig. 12**). Such cells were rarely detectable in control retina. An accompanying observation following the activation and neurogenic conversion of Müller cells in SS34ter rats was the significant but transient improvement in light perception. The evidence for Müller cell-dependent regeneration of rod photoreceptors as the cause of this improvement is indirect; first, improvement was not observed in animals in control groups. Second, retina in animals in treated groups contained activated Müller cells expressing opsin and, third, there was a strong correlation with the number of such cells with the improvement in light perception. A preservation of degenerating photoreceptors, due to the treatment and/or in response to the trophic effects exerted by activated Müller cells, as the cause for the improvement in light perception cannot be ruled out. The transitory improvement in light perception might be due to the inability of the environment to support the survival, function or both of the nascent or rescued rod photoreceptors. Taken together, our observations suggest that the stem cell properties and neurogenic potential of Müller cells are evolutionarily conserved and, hence, these cells and Notch and Wnt signaling constitute cellular and molecular targets for therapeutic regeneration of the retina.

## Supporting Information

Figure S1 Cell apoptosis after differentiation phase. Immunofluorescence analysis of sections of retinal explant from Nrl-GFP mice after differentiation in PN1CM, revealed few cleaved Caspase 3 positive cells located in the GCL. GCL =  ganglion cell layer, ONL =  outer nuclear layer.
(2.72 MB TIF)

Figure S2 Notch and Wnt signaling-mediated activation of Müller cells in rd mice explants. Retinal explants from rd mice (PN10) were cultured in the presence of Jag1+Wnt3a for 4 days followed by differentiation the examination of activation by immunofluorescence, Hoechst dye efflux and RT-PCR analyses. Immunofluorescence analysis of retinal explant section revealed the presence of BrdU+ cells co-expressing GS (arrows) (A, B) and the proportion of BrdU+GS+ cells were significantly higher in treated group, compared to control (C). Q-PCR analysis of gene expression revealed increase in levels of transcripts corresponding to Ki67, cyclD1, Hes1, Hes5 and decrease in p27kip1 transcript levels in Jag1+Wnt2b treated retina, compared to controls (D). Hoechst dye efflux assay revealed a higher proportion of SP cells (0.4%) in Jag1+Wnt2b treated retina, compared to that in controls (0.1%) (E, F). RT-PCR analysis of SP and NSP cells from Jag1+Wnt3a treated explants revealed a differential gene expression with transcripts corresponding to stem cell marker (Abcg2), neural progenitor markers (Rx and Sox2), cell cycle regulators (CyclinD1 and Ki67), and transducers of Notch (Hes1) and Wnt (Lef1) enriched in SP cells (G). In contrast, p27kip1 transcripts were enriched in NSP cells. Scale  = 20 µM *** = p<0.0001.
(7.94 MB TIF)

Figure S3 Cellular distribution of opsin immunoreactivities in cell dissociates from S334ter retina. Immunofluorescence analysis of retinal cells by confocal microscopy in different planes along z-axis reveals opsin immunoreactivities associated with cell membrane at deeper planes as compared to their apparent nuclear localization at the superficial plane. As expected, BrdU-immunoreactivities are nuclear, regardless of the plane of the axis, while those corresponding to GS and Opsin show an apparent nuclear distribution at the superficial plane.
(2.95 MB TIF)
