# Supplementary figures and images for: Notch and Wnt Signaling Mediated Rod Photoreceptor Regeneration by Müller Cells in Adult Mammalian Retina
Source: PLoS One. 2010 Aug 26;5(8):e12425. doi: 10.1371/journal.pone.0012425 (PMC2928741; doi:10.1371/journal.pone.0012425)

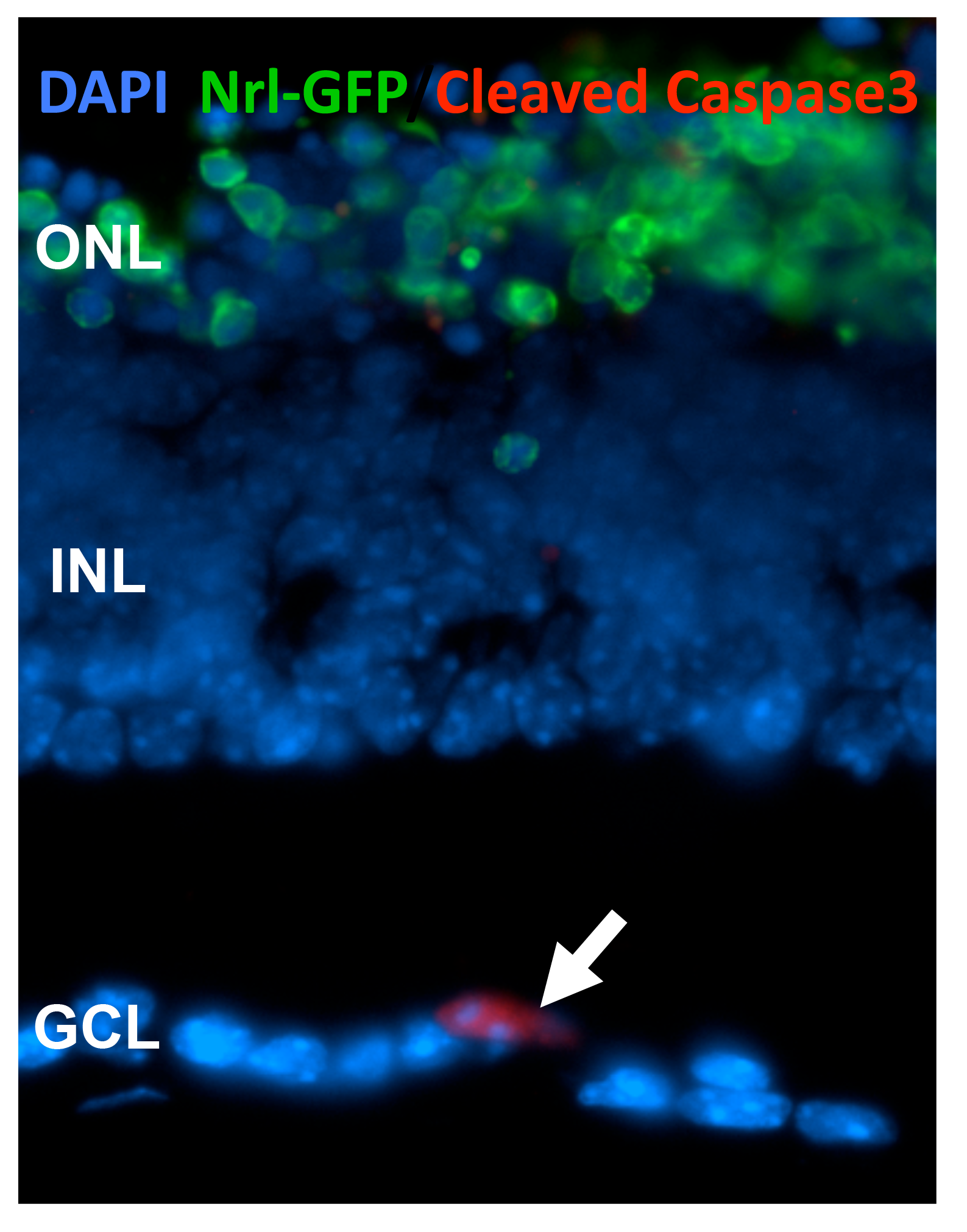

Supplement: Figure S1 — Cell apoptosis after differentiation phase. Immunofluorescence analysis of sections of retinal explant from Nrl-GFP mice after differentiation in PN1CM, revealed few cleaved Caspase 3 positive cells located in the GCL. GCL = ganglion cell layer, ONL = outer nuclear layer. (2.72 MB TIF) [file pone.0012425.s001.tif]

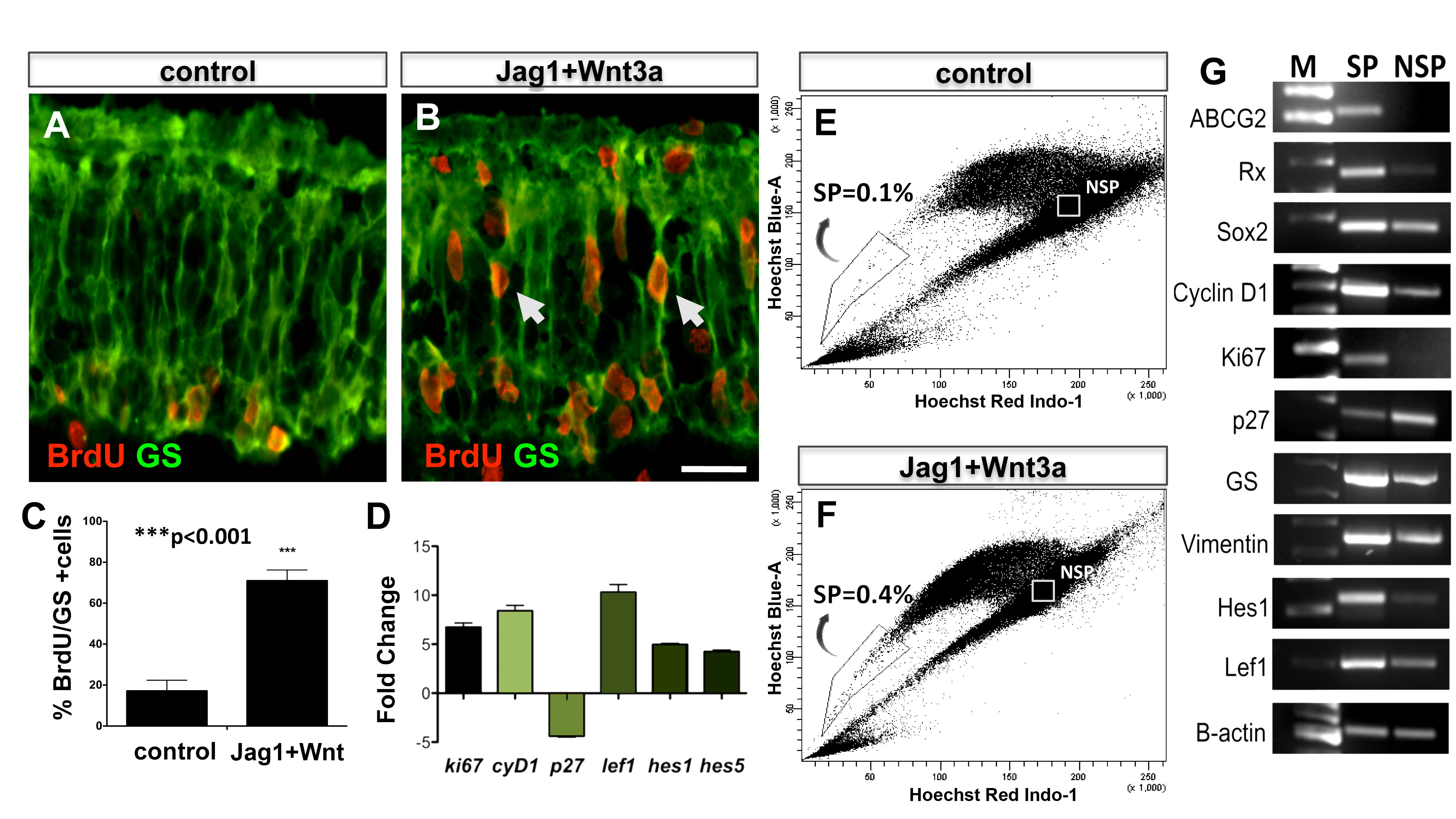

Supplement: Figure S2 — Notch and Wnt signaling-mediated activation of Müller cells in rd mice explants. Retinal explants from rd mice (PN10) were cultured in the presence of Jag1+Wnt3a for 4 days followed by differentiation the examination of activation by immunofluorescence, Hoechst dye efflux and RT-PCR analyses. Immunofluorescence analysis of retinal explant section revealed the presence of BrdU+ cells co-expressing GS (arrows) (A, B) and the proportion of BrdU+GS+ cells were significantly higher in treated group, compared to control (C). Q-PCR analysis of gene expression revealed increase in levels of transcripts corresponding to Ki67, cyclD1, Hes1, Hes5 and decrease in p27kip1 transcript levels in Jag1+Wnt2b treated retina, compared to controls (D). Hoechst dye efflux assay revealed a higher proportion of SP cells (0.4%) in Jag1+Wnt2b treated retina, compared to that in controls (0.1%) (E, F). RT-PCR analysis of SP and NSP cells from Jag1+Wnt3a treated explants revealed a differential gene expression with transcripts corresponding to stem cell marker (Abcg2), neural progenitor markers (Rx and Sox2), cell cycle regulators (CyclinD1 and Ki67), and transducers of Notch (Hes1) and Wnt (Lef1) enriched in SP cells (G). In contrast, p27kip1 transcripts were enriched in NSP cells. Scale = 20 µM *** = p<0.0001. (7.94 MB TIF) [file pone.0012425.s002.tif]

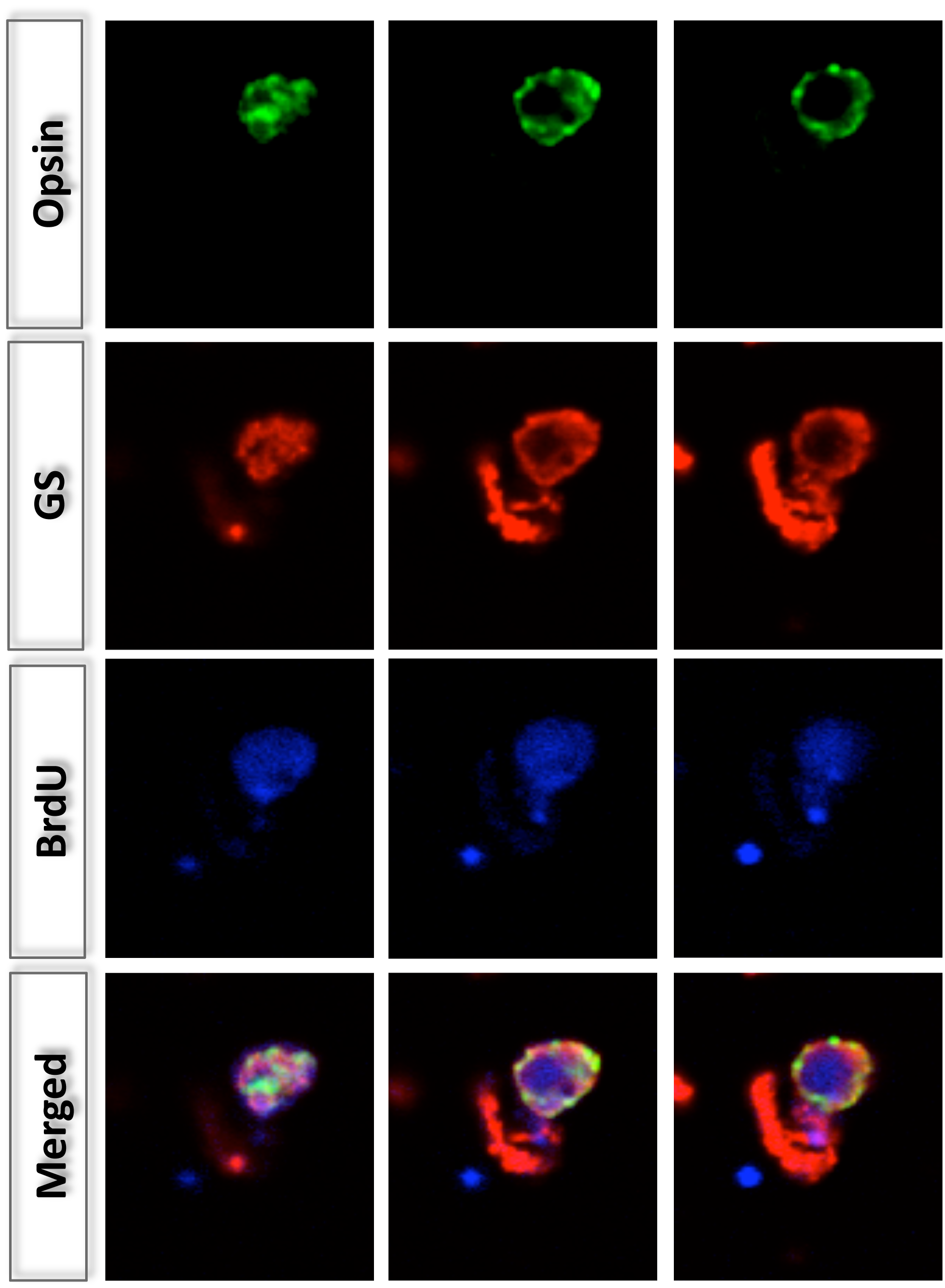

Supplement: Figure S3 — Cellular distribution of opsin immunoreactivities in cell dissociates from S334ter retina. Immunofluorescence analysis of retinal cells by confocal microscopy in different planes along z-axis reveals opsin immunoreactivities associated with cell membrane at deeper planes as compared to their apparent nuclear localization at the superficial plane. As expected, BrdU-immunoreactivities are nuclear, regardless of the plane of the axis, while those corresponding to GS and Opsin show an apparent nuclear distribution at the superficial plane. (2.95 MB TIF) [file pone.0012425.s003.tif]
